# The association between non-motor symptoms and cost in Parkinson’s disease

**DOI:** 10.1007/s00415-025-13044-w

**Published:** 2025-03-28

**Authors:** Anna Gustafsson, Frida Hjalte, Jenny Norlin, Per Odin, Peter Hagell

**Affiliations:** 1 The Swedish Institute for Health Economics, Lund, Sweden; 2 Department of Neurology, Rehabilitation Medicine, Memory Disorders, and Geriatrics, Skåne University Hospital, Malmö, Sweden; 3 SWEPAR-Net, Lund University, Lund, Sweden; 4 Restorative Parkinson Unit, Division of Neurology, Department of Clinical Sciences, Lund University, Lund, Sweden; 5 The PRO-CARE Group, Faculty of Health Sciences, Kristianstad University, SE-291 88, Kristianstad, Sweden

**Keywords:** Parkinson’s disease, Non-motor symptoms, Resource use, Cost

## Abstract

**Background:**

Parkinson’s disease (PD) is a neurodegenerative disorder associated with substantial costs that escalate as the disease progresses. Previous research has explored the relationship between disease progression, motor symptoms, and the economic burden of PD. However, there is a lack of studies focusing on the relationship between costs and non-motor symptoms (NMS).

**Objective:**

To examine the association between societal costs and NMS in individuals with PD in Sweden.

**Methods:**

Persons with idiopathic PD in the Swedish Parkinson’s disease registry from the region of Skåne with registrations of non-motor symptoms questionnaire (NMSQ) were included. Identified subjects were linked to administrative health care data registries, to estimate annual costs. A generalized linear model was used to assess the relationship between NMS and costs.

**Results:**

NMS were present in 74% (*n* = 703) of the study population, with a mean of 6.9 symptoms per observation. The number of NMS increased with disease duration, and costs were higher for those with a greater number of symptoms. Formal care costs were 3.8 times higher in observations with at least 10 NMS. Experiencing hallucinations and/or delusions was associated with an 80–94% increase in total costs, corresponding to an additional SEK 107,000–121,000 per patient year.

**Conclusions:**

Presence of NMS in PD is associated with substantial societal costs. Findings from this study highlight the necessity for comprehensive management strategies that address both motor and non-motor symptoms to potentially alleviate the burden on patients and the healthcare system.

**Supplementary Information:**

The online version contains supplementary material available at 10.1007/s00415-025-13044-w.

## Introduction

Parkinson’s disease (PD) is a progressive neurological disorder characterized by a range of motor symptoms, including bradykinesia, rigidity, tremor and postural dysfunction. PD is also associated with numerous non-motor symptoms (NMS), such as depression, anxiety, cognitive impairment, sleep disturbances, constipation, hypotension, speech difficulties, hallucinations, delusions, and pain. With time most persons with PD (PwPD) under oral levodopa therapy develop motor and non-motor symptom fluctuations and often also dyskinesias [1]. NMS can manifest at any stage of the disease and previous studies indicate that 80–100% of PwPD experience these symptoms [2–6]. Both motor [7–9] and non-motor symptoms [1, 10] significantly impact Health-Related Quality of Life (HRQoL) of PwPD. In some cases, NMS have been reported to have an even greater influence on HRQoL than motor symptoms [11]. While most PwPD experience these symptoms in the advanced stages, some NMS result in a significant management burden even in the early stages of the disease. In addition, NMS such as neuropsychiatric and behavioral features can also create a substantial burden for caregivers [12].

No curative treatments currently exist for PD. Therefore, available treatment options focus on alleviating both motor and non-motor symptoms. As the disease advances, treatments may lose efficacy [1], and patients become increasingly dependent on formal and informal caregivers [13].

PD is associated with a large economic burden, both from a societal perspective and a healthcare perspective. As PD progresses, PwPD experience increasing symptom severity, leading to escalating direct costs for pharmaceuticals, hospitalizations, and nursing home care over their lifetime [7, 14–16]. Several studies have estimated the cost of PD [7, 14, 17–30]. Although estimated total cost varies across these studies the proportion of direct versus indirect costs have typically fallen within the ranges of 60–70% and 30–40%, respectively [20, 23, 25, 26, 28, 29]. Particularly noteworthy are the costs associated with nursing homes, which account for nearly 60% of the direct costs related to managing PD [14]. Additionally, the productivity loss of PwPD and their informal caregivers represents a significant component of the indirect costs of PD [23, 28, 30]. Despite the extensive research on PD costs, few studies have focused on the association between NMS and these costs. Therefore, the aim of this study was to analyze the association between NMS and costs in PD.

## Materials and methods

### Data sources

This study was a retrospective observational analysis based on the Swedish PD registry (ParkREG), which was established in 2011 as part of the Swedish Neuro Registries (https://neuroreg.se). ParkREG includes physician-reported data on disease severity as well as patient-reported data, including HRQoL and NMS. Working status and the use of formal and informal care is available for the Skåne-cohort. The registry currently includes around 64% (n = 11,176) of the patients in contact with specialist care (according to the Swedish National Patient register). Registrations occur in connection to when a patient visit the neurologist clinic and patient-reported information can be entered from home, at a timepoint freely chosen by the patient. The aim is to have at least one registration per year, and the mean number of registrations is 3.5 per patient.

The cohort of PwPD in the Swedish PD registry residing in the region of Skåne was linked to administrative registries; the Swedish Prescribed Drugs Register (PDR) and the regional Skåne Health Care Register (SHR). PDR contains all prescribed drugs dispensed in pharmacies. The SHR covers all visits to primary care, specialized outpatient care and inpatient care (including interventions and procedures performed during that contact). The Skåne region covers approximately 13% (1.4 million) of the Swedish population.

The study was approved by the Swedish Ethical Review Authority, Lund, Sweden (Dnr 2013/374 and 2019/05791). All patients have provided their informed consent to participate in the Swedish PD registry.

### Observations

Persons diagnosed with idiopathic PD in the Skåne region between 2013 and 2019 were included in the study sample. Several observations per patient were allowed, but only one per calendar year was included to reflect annual costs. The first observation per calendar year was selected. Calendar years with deceased patients were excluded to capture annual costs.

### Variables

The non-motor symptoms questionnaire (NMSQ) is a 30-item patient-reported instrument that is used to screen for NMS in clinics. It covers ten NMS domains: gastrointestinal tract, urinary tract, sexual function, cardiovascular (including falling), apathy/attention/memory, hallucinations/delusions, depression/anxiety/anhedonia, sleep, pain (unrelated to other cause), and miscellaneous symptoms (e.g., diplopia, weight loss). The questionnaire is completed in the “yes” and “no” fashion [31]. The burden of NMS can be quantified by counting the number of “yes” responses, resulting in a total score ranging from 0 (no symptoms) to 30 (all listed symptoms present). This total can be categorized into five NMS severity groups: 0 (no NMS), 1–5 (mild), 6–9 (moderate), 10–13 (severe), and > 13 (very severe) [32].

### Cost estimates

Annual costs per patient year were calculated using a societal perspective. This includes the costs for healthcare contacts and medication, formal and informal care, costs for transportation, and productivity loss. Costs were expressed in Swedish krona (SEK), 2019 price level. Cost calculations have previously been described in more detail elsewhere [28].

### Statistical analysis

Descriptive statistics were presented as appropriate. Differences in disease duration across NMS severity groups were analysed using the Kruskal–Wallis test. The relationship between NMS and costs were assessed with a generalized linear model. Models were fitted assuming a gamma-distributed error term with a log link due to the skewed distribution of costs with a bound at zero. Candidate variables included all NMS, sex, age, and years since diagnosis. NMS with a correlation of 0.4 or higher were grouped together. The following NMS were combined, with correlation values indicated in parentheses: attention deficit with memory problems (correlation: 0.57), hallucinations with delusions (0.44), apathy with depression (0.41), vivid dreaming with REM behavior disorder (0.59), nocturia with urgency (urinary dysfunction) (0.43), and problems having sex with altered interest in sex (sexual dysfunction) (0.49). The best model was chosen based on Akaike information criteria (AIC) using backward elimination procedure. Coefficients and standard errors were transformed to the natural scale using the link function. All analyses were conducted using R version 4.1.2.

## Results

The total sample included 961 observations of 680 PwPD. Sample characteristics are presented in Table 1. The mean number of observations per person was 1.7 and the maximum was 8.

**Table 1:** Sample characteristics (n = 961)

**Variable**	
Male, n (%)	626 (65.1%)
Age, years	
Mean (SD)	69.9 (9.4)
Median (Q1-Q3)	71 (65–76)
Time since diagnosis, years	
Mean (SD)	6.7 (6.1)
Median (Q1-Q3)	5 (2–10)
Time since debut, years	
Mean (SD)	8.5 (6.1)
Median (Q1-Q3)	7 (4–12)
Hoehn and Yahr stage, n (%)	
Stage 1	283 (29.4%)
Stage 2	370 (38.5%)
Stage 3	249 (25.9%)
Stage 4–5	49 (5.1%)
Advanced treatment†, n (%)	104 (10.1%)
Number of NMS	
Mean (SD)	6.9 (6.1)
Median (Q1-Q3)	6 (0–12)
NMS severity groups, n (%)	
No NMS	254 (26.4%)
Mild (1–5 symptoms)	193 (20.1%)
Moderate (6–9 symptoms)	186 (19.4%)
Severe (10–13 symptoms)	158 (16.4%)
Very severe (> 13 symptoms)	170 (17.7%)

†Advanced treatment includes Apomorphine infusion, DBS, Duodopa, High frequent brain stimulation, and Lecigon

*SD* standard deviation, *Q*1 first quartile, *Q*3 third quartile, *NM* non-motor symptoms

Missing values by variable: Time since diagnosis (n = 30), time since debut (n = 53)

For 74% of the observations included in the study, at least one NMS was reported. The maximum number of NMS in any observation was 27. The mean number of NMS per observation was 6.9 (SD:6.1), with a median of 6 (Q1-Q3:0–12). The prevalence of NMS was similar among men and women. Across the entire sample, the most common symptoms were nocturia (51%), urgency (40%), and dizziness (39%) (Table 2). Nocturia was the most frequently reported symptom for both genders, present in 51% of male observations and 53% of female observations. Among women, the second most common NMS was feeling sad, reported in 42% of female observations compared to 30% of male observations. For men, issues with sexual function ranked as one of the most common NMS, affecting 36% of male observations and 9% of female observations.

**Table 2:** Number (%) of observations reporting each non-motor symptom according to the NMSQ (n = 961)

	n (%)
Any symptom	707 (73.6%)
Nocturia	494 (51.4%)
Urgency	388 (40.1%)
Dizziness	370 (38.5%)
Feeling sad, blue	330 (34.3%)
Restless legs syndrome	322 (33.5%)
Difficulty falling asleep	304 (31.6%)
Memory problems	299 (31.1%)
Attention deficit	295 (30.7%)
REM sleep behavior disorder	283 (29.4%)
Vivid dreaming	253 (26.3%)
Problems having sex	253 (26.3%)
Dribbling of saliva	249 (25.9%)
Anosmia, ageusia	243 (25.3%)
Constipation	234 (24.3%)
Pain	232 (24.1%)
Unsatisfactory voiding of bowel	224 (23.3%)
Altered interest in sex	208 (21.6%)
Swelling of legs	206 (21.4%)
Falling	203 (21.1%)
Difficulty swallowing	178 (18.5%)
Anxiety	175 (18.2%)
Hyperhidrosis	172 (17.9%)
Apathy	150 (15.6%)
Diplopia	136 (14.2%)
Hallucinations	119 (12.4%)
Daytime sleepiness	93 (9.7%)
Weight change	82 (8.5%)
Nausea, vomiting	69 (7.2%)
Bowel incontinence	68 (7.1%)
Delusions	42 (4.4%)

**Fig. 1:**
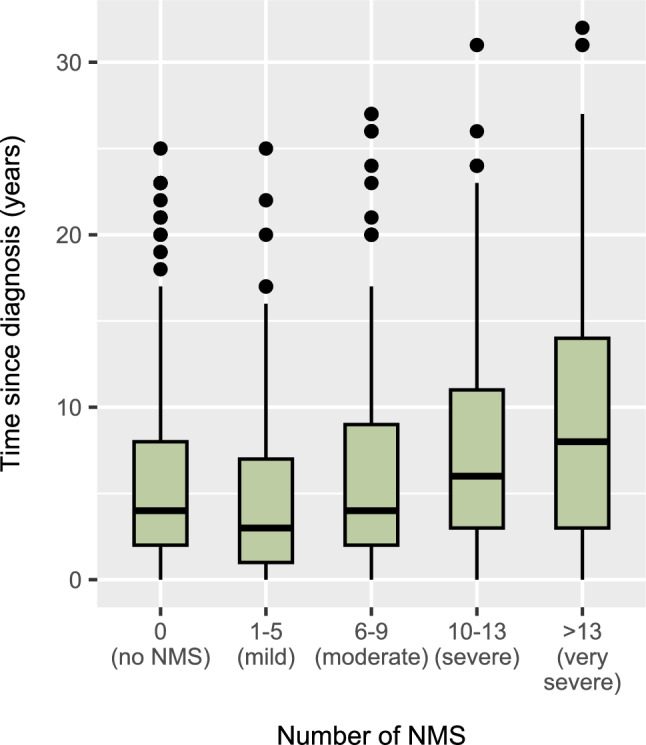
Disease duration by number of non-motor symptoms

The total number of symptoms increased with disease duration. Figure 1 shows the distribution of disease duration among the NMS severity groups, which showed a significant difference in disease duration across groups (p < 0.001). For example, the median time since diagnosis was 3 years in those with 1–5 NMS and 8 years in those with > 13 NMS. When comparing the average time since diagnosis, NMS like nocturia, nausea and vomiting, and dizziness had the lowest mean and median values, indicating that these symptoms tend to appear first. In contrast, symptoms such as delusions, diplopia, and hallucinations had the highest mean and median values, suggesting that they typically emerge later in the disease. Detailed statistics on time since PD diagnosis (years) for each non-motor symptom can be found in Online Resource 1.

**Fig. 2:**
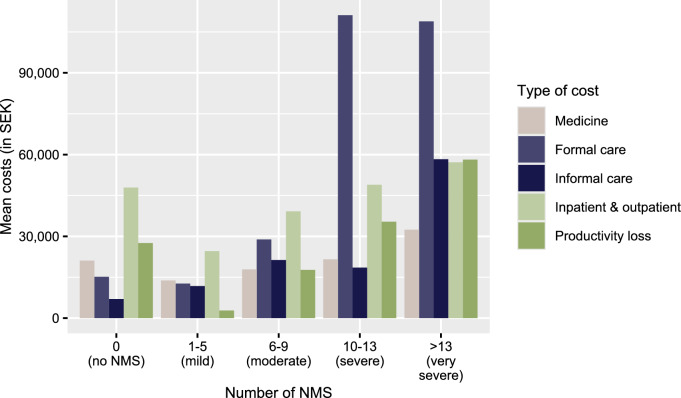
Mean cost (in SEK 2019) by type of cost and number of non-motor symptoms

The average total cost per patient year was approximately SEK 165,000. Direct costs made up 40% of the total, while indirect costs, including productivity loss, accounted for 60%. Formal care was the largest cost component, representing 77% of direct costs and 30% of the overall total. Higher costs were observed in patient years with a greater number of NMS (Fig. 2). For example, the average cost per patient-year for formal care was 3.8 times higher for observations with at least 10 NMS compared to those with 6–9 NMS.

The relationship between NMS and costs was examined through regression analysis (Table 3), which identified several NMS that significantly impact total costs. The symptoms associated with the highest cost increases were falling, hallucinations and/or delusions, nausea and vomiting, and apathy and/or depression. Specifically, costs increased by 120% for observations with falling and 80–94% for those with hallucinations and/or delusions, all else being equal. This translates to an increase in total cost per patient year of SEK 131,000–138,000 for those experiencing falling, and SEK 107,000–121,000 for those with hallucinations and/or delusions.

**Table 3:** Results from regression models of associations between non-motor symptoms and costs

	Full model	Best model
	Estimate (SE)	Estimate (SE)
Intercept	59,598.634*** (1.702)	62,551.761*** (1.678)
Male	0.931 (1.162)	0.956 (1.158)
Age	0.998 (1.007)	0.996 (1.007)
Advanced treatment†	1.697 (1.311)	1.726* (1.305)
Time since diagnosis (years)	1.077*** (1.014)	1.078*** (1.013)
Non-motor symptoms		
Dribbling of saliva	1.173 (1.190)	
Anosmia, ageusia	0.849 (1.183)	
Difficulty swallowing	0.895 (1.208)	
Nausea, vomiting	1.829* (1.310)	1.780* (1.301)
Constipation	1.190 (1.204)	
Bowel incontinence	1.212 (1.302)	
Unsatisfactory voiding of bowel	0.926 (1.207)	
Urinary dysfunction	0.892 (1.190)	
Pain	1.116 (1.186)	
Weight change	0.775 (1.282)	0.753 (1.276)
Attention deficit, memory problems	0.955 (1.193)	
Apathy, depression	1.556* (1.194)	1.574** (1.171)
Hallucinations, delusions	1.795** (1.250)	1.941** (1.233)
Anxiety	1.105 (1.225)	
Sexual dysfunction	0.887 (1.183)	0.871 (1.172)
Falling	2.200*** (1.209)	2.200*** (1.204)
Dizziness	0.922 (1.182)	
Daytime sleepiness	1.039 (1.269)	
Difficulty falling asleep	0.752 (1.177)	0.743* (1.163)
Vivid dreaming, REM behavior disorder	0.811 (1.180)	0.801 (1.171)
Restless legs syndrome	0.892 (1.183)	
Swelling	1.221 (1.196)	1.189 (1.189)
Hyperhidrosis	1.200 (1.209)	
Diplopia	0.991 (1.235)	
N	931	931
AIC	23,417	23,401

Coefficients are reported as exponentiated β and SE

† Advanced treatment includes Apomorphine infusion

*DBS* Duodopa, High frequent brain stimulation, and Lecigon

Clustred standard errors in parentheses

*** p < 0.01

** p < 0.05

* p < 0.1

## Discussion

This is one of few studies assessing the association between the non-motor symptoms and costs [14, 22]. NMS were present in 74% of the study population, and the number of symptoms increased as the disease progressed. While the overall presence of NMS was slightly lower than in previous studies [1–6, 12], the distribution and frequency of specific symptoms—such as nocturia, urgency, and dizziness—were similar to earlier findings, both in terms of which symptoms were most common and how frequently they occurred [1, 6, 12, 33–35].

The findings of this study indicate that the presence of NMS has a large impact on costs. Formal care made up 77% of direct costs and 30% of total costs, consistent with previous findings [14, 36, 37]. The cost of formal care was 3.8 times higher per patient year in observations reporting 10 NMS or more compared to those with 6–9 NMS. Results from the regression analysis indicate that hallucinations and delusions are associated with substantial costs, consistent with previous research. For instance, a study found that hallucinations were among the strongest predictors of nursing home placement for PwPD [14].

The strength of this study, based on real world data from the Swedish national registry, is the relatively large sample of 961 observations at different stages of progressed disease, reflecting everyday clinical practice. This allowed for a thorough analysis. However, it remains unclear to what extent PwPD in the Swedish PD registry represent the broader PD population. Although the sample encompasses all disease stages, it is biased towards healthier subjects. An implication of this could be a greater uncertainty in the results for the more extreme levels of severity. Another potential limitation is the use of the first NMSQ observation each calendar year in the analyses. If disease severity progressed throughout the year, this approach may have led to overestimations of costs.

Using the NMSQ to assess the presence of NMS presents some potential challenges. Firstly, the lack of information on symptom severity complicates the interpretation, as there was no available data from the Non-Motor Symptoms Scale (NMSS) or MDS-NMS. For example, we hypothesized that cognitive impairment would be associated with higher costs due to increased resource use for home care, nursing homes, and informal care. However, the questions in NMSQ related to cognitive impairment (i.e., 12. Problems remembering things that have happened recently or forgetting to do things, 13. Loss of interest in what is happening around you or doing things, and 15. Difficulty concentrating or staying focused) may only capture mild cognitive impairments, potentially explaining the lack of significant effects in our data. Secondly, due to high correlations, some NMS were grouped together, thus making it difficult to assess their individual impacts. In addition to these limitations, it should be noted that the item “falling” does not specify causes of falls (e.g., those not specifically linked to orthostatic hypertension). Consequently, the estimated costs associated with this symptom should not be interpreted as solely non-motor related.

This study examines the relationship between NMS and total costs but does not explore the extent to which these symptoms were treated. Previous research has suggested that NMS may often be undertreated [38–40]. A recent Swedish study found that, on average, only 32% of NMS were treated according to national or international guidelines [39]. Additionally, adherence to treatment guidelines varied by disease severity, with undertreatment being more common in patients with mild PD compared to those with moderate or severe PD. This could result in an underestimation of the true costs associated with symptoms that become more prevalent in the later stages of the disease, especially if those symptoms are treated more frequently than those that appear earlier. A study on compliance with treatment guidelines in late-stage PD also highlighted a gap between recommended treatments for NMS and what patients actually received [40], potentially contributing to higher overall costs due to inefficient treatment.

In conclusion, this research enhances our understanding of the economic impact of NMS in managing PD. Our findings indicate that addressing NMS should be an important objective in the management of PD. This knowledge is critical for assessing the potential value of future innovative interventions for PD, including potential disease-modifying therapies.

## Supplementary Information

Below is the link to the electronic supplementary material.

Supplementary file1 (PDF 60 KB)

## Data Availability

The data that support the findings of this study were retrieved from SWEPAR, National Board of Health and Welfare, and Skåne Region. Restrictions apply to the availability of these data, which were used under license for this study. Data are not publicly available and will not be available without permission from SWEPAR, National Board of Health and Welfare, and Skåne Region.

## References

[1] Martinez-Martin P, Rodriguez-Blazquez C, Kurtis MM, Chaudhuri KR, Group NV (2011) The impact of non-motor symptoms on health-related quality of life of patients with Parkinson’s disease. Mov Disord 26(3):399–406 21264941 10.1002/mds.23462

[2] Martinez-Martin P, Falup Pecurariu C, Odin P, van Hilten JJ, Antonini A, Rojo-Abuin JM et al (2012) Gender-related differences in the burden of non-motor symptoms in Parkinson’s disease. J Neurol 259(8):1639–1647 22237822 10.1007/s00415-011-6392-3

[3] Donzuso G, Cicero CE, Vinciguerra E, Sergi R, Luca A, Mostile G et al (2023) Gender differences in non-motor fluctuations in Parkinson’s disease. J Neural Transm (Vienna) 130(10):1249–1257 37526768 10.1007/s00702-023-02679-6 PMC10480257

[4] Barone P, Antonini A, Colosimo C, Marconi R, Morgante L, Avarello TP et al (2009) The PRIAMO study: a multicenter assessment of nonmotor symptoms and their impact on quality of life in Parkinson’s disease. Mov Disord 24(11):1641–1649 19514014 10.1002/mds.22643

[5] Fernandes M, Pierantozzi M, Stefani A, Cattaneo C, Bonizzoni EA, Cerroni R et al (2021) Frequency of non-motor symptoms in Parkinson’s patients with motor fluctuations. Front Neurol 12:678373 34267721 10.3389/fneur.2021.678373 PMC8276257

[6] Sardar Z, Liaquat S, Yousaf Q, Bano S, Numan A (2023) Non-motor symptoms burden in early stages of Parkinson’s disease. Ann Indian Acad Neurol 26(1):39–43 37034035 10.4103/aian.aian_483_22 PMC10081544

[7] Keranen T, Kaakkola S, Sotaniemi K, Laulumaa V, Haapaniemi T, Jolma T et al (2003) Economic burden and quality of life impairment increase with severity of PD. Parkinsonism Relat Disord 9(3):163–168 12573872 10.1016/s1353-8020(02)00097-4

[8] Dodel RC, Berger K, Oertel WH (2001) Health-related quality of life and healthcare utilisation in patients with Parkinson’s disease: impact of motor fluctuations and dyskinesias. Pharmacoeconomics 19(10):1013–1038 11735671 10.2165/00019053-200119100-00004

[9] Hechtner MC, Vogt T, Zollner Y, Schroder S, Sauer JB, Binder H et al (2014) Quality of life in Parkinson’s disease patients with motor fluctuations and dyskinesias in five European countries. Parkinsonism Relat Disord 20(9):969–974 24953743 10.1016/j.parkreldis.2014.06.001

[10] Martinez-Martin P (2014) Nonmotor symptoms and health-related quality of life in early Parkinson’s disease. Mov Disord 29(2):166–168 24375626 10.1002/mds.25799

[11] Berganzo K, Tijero B, González-Eizaguirre A, Somme J, Lezcano E, Gabilondo I et al (2016) Motor and non-motor symptoms of Parkinson’s disease and their impact on quality of life and on different clinical subgroups. Neurologia 31(9):585–591 25529173 10.1016/j.nrl.2014.10.010

[12] Kumar A, Patil S, Singh VK, Pathak A, Chaurasia RN, Mishra VN et al (2022) Assessment of non-motor symptoms of Parkinson’s disease and their impact on the quality of life: an observatiobnal study. Ann Indian Acad Neurol 25(5):909–915 36561034 10.4103/aian.aian_647_21 PMC9764934

[13] Chaudhuri KR, Azulay JP, Odin P, Lindvall S, Domingos J, Alobaidi A et al (2024) Economic burden of Parkinson’s disease: a multinational, real-world, Cost-of-Illness Study. Drugs Real World Outcomes 11(1):1–11 38193999 10.1007/s40801-023-00410-1 PMC10928026

[14] Hermanowicz N, Edwards K (2015) Parkinson’s disease psychosis: symptoms, management, and economic burden. Am J Manag Care 21(10 Suppl):s199-206 26296199

[15] Dahodwala N, Li P, Jahnke J, Ladage VP, Pettit AR, Kandukuri PL et al (2021) Burden of Parkinson’s disease by severity: health care costs in the U.S. medicare population. Mov Disord 36(1):133–142 33031604 10.1002/mds.28265

[16] Kellerborg K, Norlin JM, Odin P (2023) The relationship between PDQ-8 and costs in Parkinson’s disease-a Swedish Register-Based Study. Mov Disord Clin Pract 10(2):231–237 36825055 10.1002/mdc3.13630 PMC9941938

[17] Albarmawi H, Zhou S, Shulman LM, Gandhi AB, Johnson A, Myers DE et al (2022) The economic burden of Parkinson disease among Medicare beneficiaries. J Manag Care Spec Pharm 28(4):405–414 35332791 10.18553/jmcp.2022.28.4.405 PMC10372956

[18] Boland DF, Stacy M (2012) The economic and quality of life burden associated with Parkinson’s disease: a focus on symptoms. Am J Manag Care 18(7 Suppl):S168–S175 23039865

[19] Findley LJ (2007) The economic impact of Parkinson’s disease. Parkinsonism Relat Disord 13(Suppl):S8-s12 17702630 10.1016/j.parkreldis.2007.06.003

[20] Findley LJ, Wood E, Lowin J, Roeder C, Bergman A, Schifflers M (2011) The economic burden of advanced Parkinson’s disease: an analysis of a UK patient dataset. J Med Econ 14(1):130–139 21235405 10.3111/13696998.2010.551164

[21] Lökk J, Borg S, Svensson J, Persson U, Ljunggren G (2012) Drug and treatment costs in Parkinson’s disease patients in Sweden. Acta Neurol Scand 125(2):142–147 21470194 10.1111/j.1600-0404.2011.01517.x

[22] Vossius C, Larsen JP, Janvin C, Aarsland D (2011) The economic impact of cognitive impairment in Parkinson’s disease. Mov Disord 26(8):1541–1544 21538519 10.1002/mds.23661

[23] Yang JX, Chen L (2017) Economic burden analysis of Parkinson’s disease patients in China. Parkinsons Dis 2017:8762939 28695039 10.1155/2017/8762939 PMC5488490

[24] Zhao YJ, Tan LC, Au WL, Heng DM, Soh IA, Li SC et al (2013) Estimating the lifetime economic burden of Parkinson’s disease in Singapore. Eur J Neurol 20(2):368–374 22978629 10.1111/j.1468-1331.2012.03868.x

[25] von Campenhausen S, Winter Y, Silva AR, Sampaio C, Ruzicka E, Barone P et al (2011) Costs of illness and care in Parkinson’s disease: an evaluation in six countries. Eur Neuropsychopharmacol 21(2):180–191 20888737 10.1016/j.euroneuro.2010.08.002

[26] Winter Y, Balzer-Geldsetzer M, Spottke A, Reese JP, Baum E, Klotsche J et al (2010) Longitudinal study of the socioeconomic burden of Parkinson’s disease in Germany. Eur J Neurol 17(9):1156–1163 20345926 10.1111/j.1468-1331.2010.02984.x

[27] Spottke AE, Reuter M, Machat O, Bornschein B, von Campenhausen S, Berger K et al (2005) Cost of illness and its predictors for Parkinson’s disease in Germany. Pharmacoeconomics 23(8):817–836 16097843 10.2165/00019053-200523080-00007

[28] Hjalte F, Norlin JM, Kellerborg K, Odin P (2021) Parkinson’s disease in Sweden-resource use and costs by severity. Acta Neurol Scand 144(5):592–599 34254292 10.1111/ane.13502

[29] Tamás G, Gulácsi L, Bereczki D, Baji P, Takáts A, Brodszky V et al (2014) Quality of life and costs in Parkinson’s disease: a cross sectional study in Hungary. PLoS ONE 9(9):e107704 25229404 10.1371/journal.pone.0107704 PMC4167855

[30] Hagell P, Nordling S, Reimer J, Grabowski M, Persson U (2002) Resource use and costs in a Swedish cohort of patients with Parkinson’s disease. Mov Disord 17(6):1213–1220 12465059 10.1002/mds.10262

[31] Chaudhuri KR, Martinez-Martin P, Schapira AH, Stocchi F, Sethi K, Odin P et al (2006) International multicenter pilot study of the first comprehensive self-completed nonmotor symptoms questionnaire for Parkinson’s disease: the NMSQuest study. Mov Disord 21(7):916–923 16547944 10.1002/mds.20844

[32] Chaudhuri KR, Sauerbier A, Rojo JM, Sethi K, Schapira AH, Brown RG et al (2015) The burden of non-motor symptoms in Parkinson’s disease using a self-completed non-motor questionnaire: a simple grading system. Parkinsonism Relat Disord 21(3):287–291 25616694 10.1016/j.parkreldis.2014.12.031

[33] Gökçal E, Gür VE, Selvitop R, Babacan Yildiz G, Asil T (2017) Motor and non-motor symptoms in Parkinson’s disease: effects on quality of life. Noro Psikiyatr Ars 54(2):143–148 28680312 10.5152/npa.2016.12758 PMC5491664

[34] Diaconu Ş, Irincu L, Ungureanu L, Țînț D, Falup-Pecurariu C (2023) Nocturia and sleep in Parkinson’s disease. J Pers Med 13(7):1053 37511666 10.3390/jpm13071053 PMC10381144

[35] Rahman SS, Acherjya GK, Ali M, Alam MS, Mondal G, Saha K et al (2023) Assessment of the relationship between non-motor features and severity of Parkinson’s disease patients in Bangladesh. Mymensingh Med J 32(2):463–475 37002759

[36] Yang W, Hamilton JL, Kopil C, Beck JC, Tanner CM, Albin RL et al (2020) Current and projected future economic burden of Parkinson’s disease in the U.S. NPJ Parkinsons Dis 6:15 32665974 10.1038/s41531-020-0117-1 PMC7347582

[37] Kowal SL, Dall TM, Chakrabarti R, Storm MV, Jain A (2013) The current and projected economic burden of Parkinson’s disease in the United States. Mov Disord 28(3):311–318 23436720 10.1002/mds.25292

[38] Baig F, Lawton M, Rolinski M, Ruffmann C, Nithi K, Evetts SG et al (2015) Delineating nonmotor symptoms in early Parkinson’s disease and first-degree relatives. Mov Disord 30(13):1759–1766 26179331 10.1002/mds.26281 PMC5034839

[39] Janz C, Timpka J, Rosqvist K, Paul G, Storch A, Odin P (2024) Non-motor symptom management: insights into adherence to treatment guidelines in Parkinson’s disease patients. J Parkinsons Dis 14(2):297–312 38217612 10.3233/JPD-230263 PMC10977407

[40] Rosqvist K, Odin P (2023) Compliance with national and international guidelines in the treatment of nonmotor symptoms in late-stage Parkinson’s disease. Parkinsons Dis 2023:6667339 37854895 10.1155/2023/6667339 PMC10581854

