# Supplementary material for: The association between non-motor symptoms and cost in Parkinson’s disease
Source: J Neurol. 2025 Mar 28;272(4):297. doi: 10.1007/s00415-025-13044-w (PMC11953211; doi:10.1007/s00415-025-13044-w)
Supplement: Supplementary file 1 — Supplementary file1 (PDF 60 KB) [file 415_2025_13044_MOESM1_ESM.pdf]

The association between non-motor symptoms and cost in Parkinson's Disease, Journal of Neurology

Anna Gustafsson MSc [a], Frida Hjalte MSc[a], Jenny Norlin, PhD[a], Per Odin MD PhD [b, c, d], Peter Hagell RN PhD [d, e]

[a] The Swedish Institute for Health Economics, Lund, Sweden.

[b] Department of Neurology, Rehabilitation Medicine, Memory Disorders, and Geriatrics, Skåne University Hospital, Malmö, Sweden

[c] SWEPAR-net

[d] Restorative Parkinson Unit, Division of Neurology, Department of Clinical Sciences, Lund University, Lund, Sweden

[e] The PRO-CARE Group, Faculty of Health Sciences, Kristianstad University, Kristianstad, Sweden.

Corresponding author

Peter Hagell, peter.hagell@hkr.se

Supplementary information

Table 1 Time since PD diagnosis (years) for each non-motor symptom

Non-motor symptoms	Mean (SD)	Median (Q1-Q3)
Nocturia	7 (6.1)	5 (2-11)
Nausea, vomiting	7 (5.9)	5 (2-10)
Dizziness	7.1 (6.1)	5 (2-10)
Anxiety	7.1 (6.3)	5 (2-11)
Decreased libido	7.3 (6.7)	5 (2-11)
Restless legs syndrome (RLS)	7.4 (6.1)	6 (3-11)
Insomnia	7.4 (6.2)	5 (2-12)
Memory problems	7.5 (6.4)	6 (3-11)
Pain	7.5 (6.3)	5 (2-11.5)
Apathy	7.6 (6.6)	6 (2-11)
Weight loss	7.6 (6.4)	6 (3-11)
Feeling sad, blue	7.7 (6.5)	6 (2-12)
Swelling	7.8 (6.6)	6 (2-11)
Unsatisfactory voiding of bowel	7.9 (6.6)	6 (2-11.5)
Constipation	7.9 (6.2)	6 (3-11)
Anosmia, ageusia	7.9 (6.6)	6 (3-12)
Vivid dreaming	8 (6.4)	6 (3-12)
Urgency	8.1 (6.5)	6 (3-12)
Attention deficit	8.1 (6.8)	6 (3-12)
REM behavior disorder (RBD)	8.3 (6.5)	6 (3-12)
Sexual function	8.4 (6.7)	7 (3.5-12)
Fecal incontinence	8.5 (7.2)	6 (2-13)
Hyperhidrosis	8.7 (6.7)	7 (3-12.5)
Excessive daytime sleepiness (EDS)	8.8 (6.8)	7 (4-12)
Difficulty swallowing	8.9 (7.5)	7 (3-13)
Dribbling of saliva	9 (7.4)	7 (3-13.5)
Falling	9.7 (7.3)	8 (4-14.5)
Hallucinations	10.1 (7.2)	9 (4-14)
Diplopia	10.2 (6.8)	9 (5-13)
Delusions	11.6 (8.3)	9 (5-18)
